# Nanocellulose and Polycaprolactone Nanospun Composite Membranes and Their Potential for the Removal of Pollutants from Water

**DOI:** 10.3390/molecules25030683

**Published:** 2020-02-06

**Authors:** Hasbleidy Palacios Hinestroza, Hilary Urena-Saborio, Florentina Zurita, Aida Alejandra Guerrero de León, Gunasekaran Sundaram, Belkis Sulbarán-Rangel

**Affiliations:** 1 Department of Water and Energy, University of Guadalajara Campus Tonalá, Tonalá 45425, Mexico; hasble27@yahoo.es (H.P.H.); aida.guerrero@academico.udg.mx (A.A.G.d.L.); 2 Food and Bioprocess Engineering Lab, Department of Biological Systems Engineering, University of Wisconsin, Madison, WI 53706, USA; hurena@wisc.edu (H.U.-S.); guna@wisc.edu (G.S.); 3 Quality Environmental Laboratory, University of Guadalajara Campus Ciénega, Ocotlán 47829, Mexico; fzurita@cuci.udg.mx

**Keywords:** agave bagasse, cellulose nanofibers, membranes, electro-spinning, water filtration

## Abstract

A composite membrane based on polycaprolactone (PCL) and cellulose nanofibers (CNF) with different compositions was prepared using the electro-spinning method, with the objective of developing organic membranes with good mechanical properties to remove contaminants from water. Water is a resource of primary importance for life and human activities. In this sense, cellulose obtained from agave bagasse and polycaprolactone nanofibers was used to prepare membranes that were tested by filtering tap water. The membranes obtained presented a porosity and structure on a nanometric scale. The water quality variables evaluated after filtration with the PCL/CNF membranes showed 100% turbidity removal, 100% conductivity, and heavy metal removal of the order of 75% to 99% for iron and chromium. CNF comprises biowaste derived from tequila production, and it has added value. Electro-spun CNF and PCL membranes can be applied as a “green” and eco-friendly filtration system for water purification.

## 1. Introduction

The World Health Organization (WHO), in collaboration with the United Nations Educational, Scientific and Cultural Organization (UNESCO), has established water and sanitation as being integral components in the sustainable development agenda [1]. It was decreed that the availability of water resources must be guaranteed in the decades to come, since they are key to guaranteeing life on Earth [2,3]. Tap water must meet regulatory standards for consumption; however, in countries such as Mexico, this does not happen [4]. This is because they do not have the necessary infrastructure for purification of the water that comes out of taps in homes; tap water can contain some pollutants. In addition, in some places, the pipes that transport water to homes are in poor condition and contain metal, discoloration, or chemical contaminants in the distribution system [5]. For this reason, the development of new organic/green technologies that allow the elimination of pollutants in tap water has become very important. This has led to efforts in developing nanostructural membranes for potential applications in water treatment [6]. 

Agave bagasse is an abundant source of lignocellulosic biomass [7,8,9,10], produced mainly in the state of Jalisco, Mexico. This solid, fibrous by-product is obtained after grinding and extracting the fermentable sugars from agave pineapples during tequila production [10]. This biomass represents about 40% (wet basis) of over 1000 tons of agave grown in Mexico [11]. The availability of such large quantities of agave bagasse [9] poses serious disposal problems, since most of the biomass ends up in clandestine dumps (due to little environmental regulation), causing adverse effects on the fertility of farmland [12], contamination by leachates, and phytosanitary risks due to the inadequate incorporation of this material into the soil [13]. Chemically, agave bagasse is 44.5% cellulose, 25.3% hemicellulose, and 20.1% lignin [7]. Taking advantage of these constituents, there have been many efforts to use agave bagasse for various applications, e.g., the production of biopolymers [14,15], composting [16], animal nutrition [17], generation of biofuels [10,18], reinforcement materials [19], etc. More recently, agave bagasse is being explored for the production of cellulose nanofibers and nanocrystals [7,8,20,21] to further diversify the utilization of this biowaste. On the other hand, electro-spinning of biopolymers has become popular in the manufacture of nanofibers for a variety of added value applications, such as tissue engineering, drug release, sensors, and air and liquid filtration systems [22,23,24,25,26]. 

Our main interest in electro-spinning agave cellulose nanofibers was to produce membranes that can retain suspended particles in water (due to the diameter of the fibers), a membrane with high porosity and good surface area properties. However, the main challenge with the preparation of electro-spun agave cellulose was the poor solubility of cellulose that was to be dispersed into an electro-spinnable liquid [24]. We overcame the poor solubility challenge by dissolving agave cellulose in dimethylformamide (DMF) and blending with polycaprolactone to prepare a composite electro-spun blend, subsequently producing electro-spun nanocellulose and polycaprolactone membranes. The main objective of using a copolymer blend was to improve technical properties, such as contaminant retention capacity, thermal stability, mechanical resistance, and chemical properties [24]. In this way, cellulose and polycaprolactone composites allow the preparation of a green and eco-friendly material from agave biowaste for its use in filtration systems. To date, the production of electro-spun agave cellulose and polycaprolactone nanofiber membranes has not yet been reported in literature. We then prepared electro-spun nanofibers of agave cellulose and polycaprolactone to produce membranes for water filtration.

On the other hand, domestic water filtration systems are widely used throughout the world, since they are very effective. These household filter systems consist of placing a filter at the tap outlet and using the water pressure that comes out of the pipe [26,27,28]. These filters are made of a variety of different materials, such as ceramics, activated carbon, and synthetic polymers [26]. Such materials are not biodegradable and generate another residue in the environment [27]. Therefore, the objective of this research is to prepare electro-spun, organic membranes from agave bagasse and polycaprolactone that have the ability to be used in the process of the filtration of tap water. The membranes can be used for selective contaminants (such as heavy metals in a colloidal state) and also reduce turbidity and conductivity of the water used for human consumption. It has been reported that cellulose and nanocellulose are effective in the adsorption of metals, such as cadmium, nickel, and silver, due to its negative surface charge [29]. In addition, the crystallinity and surface area of the cellulose (at nanometer scale) are enhanced, and other properties of interest also increase. Composite materials of nanocellulose and other synthetic polymers have also been reported and are shown to adsorb other metals, such as iron, chromium, and cadmium [6,30], due to surface loading. It is expected that electro-spun agave bagasse and polycaprolactone membranes separate the solids according to their sizes and retain certain metal ions due to the negative surface charges in their functional groups [7]. 

## 2. Results and Discussion

### 2.1. Characterization of the Electro-spun Agave Bagasse Membranes

**Figure 1:**
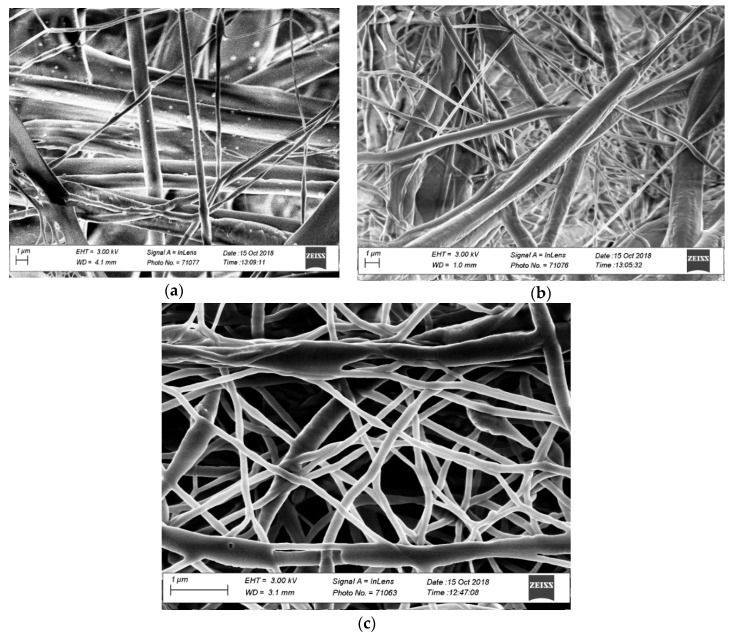
SEM images of electro-spun nanofibrous membranes fabricated from polycaprolactone/cellulose nanofiber (PCL/CNF) copolymer blends at (*v*/*v*) ratios: (**a**) 80:20, (**b**) 60:40, and (**c**) 50:50.

The SEM micrographs (Figure 1) show the morphology of electro-spun nanofibers constituting the membranes. In general, all the membranes showed high porosity and pore interconnectivity, which could promote the filtration of water and adsorption of contaminants. 

The average diameter of electro-spun nanofibers is affected by the composition of the PCL/CNF blend used for electro-spinning (Table 1). The average (min, max) diameter of the electro-spun nanofibers formed with PCL80:CNF20, PCL60:CNF40, and PCL50:CNF50 blends were 1049 nm (150 nm, 2000 nm); 1747 nm (120 nm, 3500 nm); and 216 nm (40 nm, 250 nm), respectively. This suggests that the incorporation of higher percentages of PCL (60% and 80%) may increase the viscosity of the copolymer blend, which, in turn, tends to increase the average diameters of the electro-spun nanofibers [31,32,33]. Table 1 also shows the porosity and permeability values of the membranes, which depends on the morphology of the fibers and the interconnection between them. The membrane of PCL80:CNF20 is more permeable than PCL60:NFC40 and PCL50:NFC50. Permeability is the ability of the membrane to allow a flow through the pores of its structure over a given time, and it is an important feature of the filtration process. The PCL60:CNF40 and PCL50:CNF50 membranes had the same permeability but different porosity. By having fibers of a nanometric scale, the PCL50:CNF50 membrane has pores that are smaller than the other membranes. In comparison to commercial cellulose membranes, they have greater permeability and porosity. 

**Figure 2:**
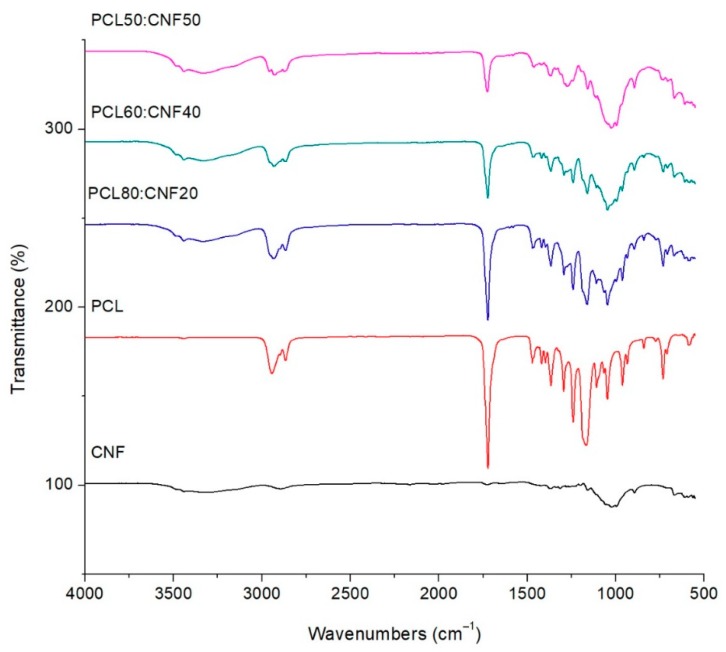
FTIR spectra of cellulose nanofibers (CNF), polycaprolactone (PCL), and electro-spun membranes for 50:50, 60:40, and 80:20 PCL/CNF (*v*/*v*) ratios.

Another important aspect of contaminant retention is the functional groups that may be present in the membranes, because these could interact with the contaminants. FTIR analysis was performed for the membranes, and the FTIR spectroscopy data (Figure 2) showed absorbance intensities at 3335, 2895, 1495, 1140, 1060, and 905 cm^−1^ for the CNF starting material; these are associated with pure cellulose [34]. The adsorption at 3335 cm^−1^ is due to the stretching of hydroxyl groups (OH) and, at 2895 cm^−1^, is due to C–H stretching. The peaks at 1140 and 1060 cm^−1^ are attributed to C=O, C–C, and C–O groups, and the peaks at 905 cm^−1^ correspond to C–O–C bending [7]. 

In the PCL spectrum (Figure 2), we can easily identify strong bands, such as carbonyl (C=O), stretching around 1723 cm^−1^. The peaks at 2954 and 2859 cm^−1^ correspond to the asymmetric and symmetric CH_2_ stretching. The bands at 1473, 1243, and 1176 cm^−1^ are related to the C–C bending, asymmetric C–O–C stretching, and symmetric C–O–C stretching. The band at 1291 cm^−1^ corresponds to C–O and C–C stretching in the crystalline phase, and the band at 1157 cm^−1^ corresponds to C–O and C–C stretching in the amorphous phase [34]. In the spectrum of the PCL/CNF composite membranes, the characteristic peak for PCL at 1723 cm^−1^ shows a decrease in the intensity function to the CNF added to the copolymer blend, which suggests a decrease of the carbonyl group. Similarly, the pronounced signal at 3335 cm^−1^, corresponding to the OH groups present in CNF, seems to increase its intensity as the CNF content increases in the copolymer blend with PCL. Each membrane that includes cellulose fiber contains reactive OH groups, which could generate hydrogen bonds. Hydrogen bonding plays an important role in the adsorption process for the specific bonding that originates on the bonding sites of the composites [29].

**Figure 3:**
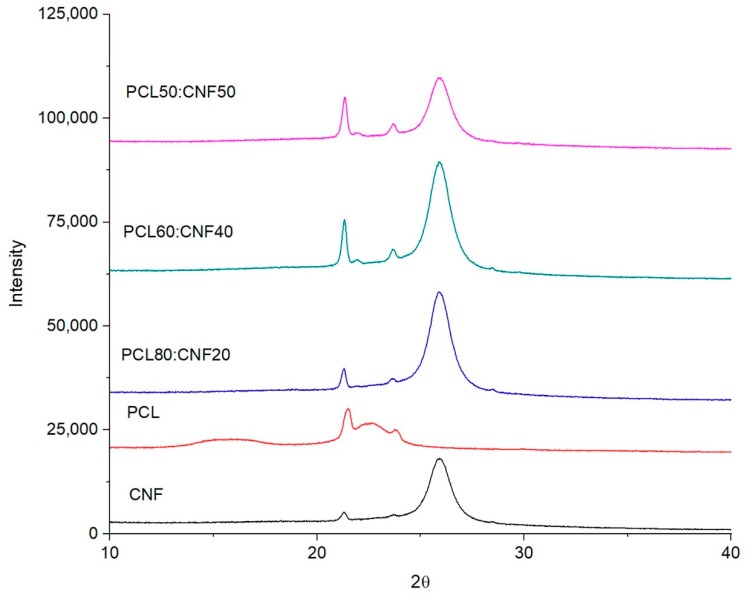
XRD spectra of cellulose nanofibers (CNF), polycaprolactone (PCL), and electro-spun membranes of 50:50, 60:40, and 80:20 PCL/CNF (*v*/*v*) ratios.

The accessibility of the OH groups on the surface of the membranes is affected by the degree of crystallinity of the composite. According to this, the degree of crystallinity was measured and the crystalline planes of the membranes determined by XRD. The XRD spectrum (Figure 3) showed characteristic 2 theta peaks for PCL at 21.5°and 23.8°, corresponding to the planes (110) and (200), respectively. This is due to the semi-crystalline structure of PCL [34]. Similarly, the spectrum of CNF shows a maximum 2 theta peak at 25°, corresponding to the (101) plane. On the other hand, the spectra of PCL/CNF electro-spun membranes showed a composition with a decrease in the intensity of the peaks at 25°, 23.8°, and 21.5°, which directly correlated to the increase in CNF content. The crystallinity percentages calculated from the XRD spectra of CNF and PCL/CNF membranes correspond to 68.5% and 71.7%, respectively [8]. 

**Figure 4:**
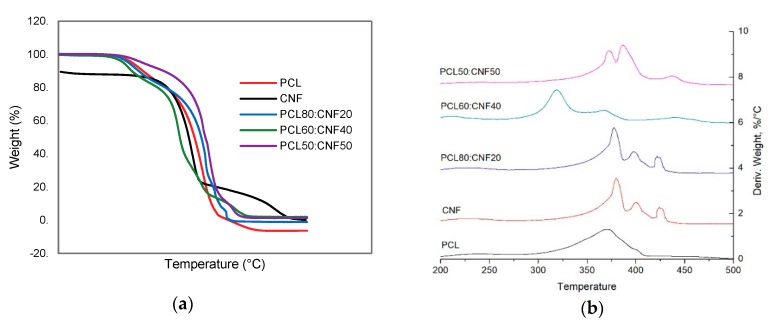
Thermogravimetric analysis: (**a**) TGA curves of cellulose nanofibers (CNF), polycaprolactone (PCL), and electro-spun membranes of 50:50, 60:40, and 80:20 PCL/CNF (*v*/*v*) ratios. (**b**) DTG curves of cellulose nanofibers (CNF), polycaprolactone (PCL), and electro-spun membranes of 50:50, 60:40, and 80:20 PCL/CNF (*v*/*v*) ratios.

Thermogravimetric analysis (TGA) was performed to investigate the thermostability of the PCL/CNF membranes in comparison to those of CNF and PLC. Figure 4 shows that a major weight loss occurs between 200 and 400 °C in the PCL/CNF membranes. The remnant weight percentage for the single PCL and CNF was about 5% and 25%, respectively. Initially, the TG curves for CNF show a small drop between 50 and 100 °C, which corresponds to a mass loss of approximately 5%, due to the moisture absorbed. Then, there was a weight loss event at approximately 343 °C, corresponding to a 78% weight loss that can be attributed to the depolymerization of CNF. PCL and PCL/CNF membranes exhibited similar thermal degradation profiles, showing a loss of mass in the range of 200 to 430 °C. This event was ascribed to polymer chain decomposition. The thermal stability of all the samples was analyzed by comparing the 10% (T10) and 50% (T50) weight-loss temperatures. The T10 and T50 of PCL started at 358 and 390 °C, respectively, while all CNF showed higher T10 (341°) and T50 (503°) values. The addition of PCL50:CNF50 increased the thermal stability of the PCL to 380° and 429 °C for T10 and T50, respectively (see the DTG curve, Figure 4b). This behavior can be attributed to the interaction between the interface of CNF and PCL, which increases the interaction between the polymer chains; this is in agreement with previous studies reported in the literature [35].

**Figure 5:**
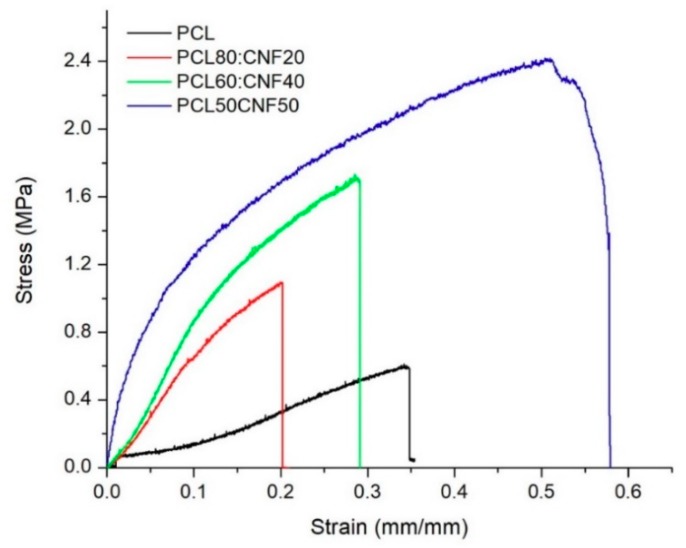
Stress–strain curves of polycaprolactone (PCL) and electro-spun membranes of 50:50, 60:40, and 80:20 PCL/CNF (*v*/*v*) ratios.

The mechanical properties of the electro-spun membranes are important for their uses in water filtration, since they must withstand the pressure exerted by flowing water. The typical stress-strain curves of PCL and PCL/CNF composite, electro-spun membranes, are shown in Figure 5. It was observed that in electro-spun membranes with the mixture of PCL/CNF at 80:20 and 60:40, the lowest elongation break values (0.21 and 0.30 mm/mm) were presented, respectively. The electro-spun membranes of pure PCL presented a greater elongation break value (0.36 mm/mm). The decrease in elongation at the point of rupture that was observed in the mixtures of PCL: NFC at 80:20 and 60:40 can be attributed to a low interaction between PCL and NFC, which is usually indicative of the immiscibility between components in the mixture when the pure polymer is used [36]. However, PCL/CNF (50:50) composite membrane showed the highest elastic modulus and tensile strength (0.58 mm/mm) of the three membrane systems that were fabricated. The increase in the elastic modulus and tensile strength could be due to a more effective interaction between CNF and PCL in the composite blend, resulting in an enhanced stiffness of the biomaterial.

### 2.2. Filtration Performance of the PCL:CNF Composite Electro-spun Membranes

**Figure 6:**
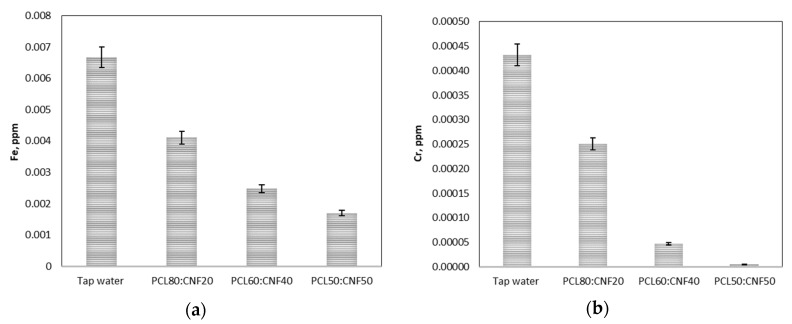
Removal of heavy metals in tap water using PCL/CNF (*v*/*v*) ratio (80:20, 60:40, and 50:50) composite electro-spun membranes: (**a**) Iron (Fe) and (**b**) Chromium (Cr).

The filtration performance of the CNF:PCL composite electro-spun membrane was tested using tap water. Tap water must meet regulatory standards for consumption; however, in some places, the pipes that transport water to homes are in poor condition, introducing metal, discoloration, or chemical contaminants into the distribution system. Tap water was analyzed before filtration to look at the types of pollution they contained. Using atomic absorption spectrophotometry, it was possible to determine the heavy metal content; we found concentrations of iron and chrome of 0.006675857 and 0.000432294 ppm, respectively. After filtration of the tap water with the electro-spun membranes composed of PCL and NFC, there was a decrease in the concentration of iron and chromium in the filtered water, as seen in Figure 6.

If the reduction of heavy metals is analyzed in terms of percentage (Table 2), in the case of Fe, it was 38%, 62%, and 75% using the PCL80:CNF20, PCL60:CNF40, and PCL50:CNF50 membranes, respectively. Whereas, for Cr, the removal percentage was higher, showing reductions of 42%, 90%, and 99% for the PCL80:CNF20, PCL60:CNF40, and PCL50:CNF50 membranes, respectively. The retention of these heavy metals by electro-spun membranes is mainly attributed to exclusion by pore size, since these metals were probably in colloidal form. Another reason for the retention could be explained by the interactions between the functional sites of the PCL and CNF electro-spun membrane surface with the heavy metal ions [36,37]. Hydrogen bonding played an important role in the adsorption process for the specific bonding that originates on the bonding sites of the composites, and, for that reason, the cellulose nanofibers were the active material in the composite because their surfaces possess different functional groups. Each unit of D-glucopyranoside within the cellulose chain contains three reactive OH groups, two secondaries (OH-C_2_ and OH-C_3_), and one primary (OH-C_6_). These negative hydroxyl groups, present in cellulose nanofibers [7], were able to form bonding groups with the positively charged Fe and Cr metal ions [30,38]. This was observed in membranes with a higher percentage of cellulose nanofibers (PCL50:CNF50), where greater retention of heavy metals was obtained compared to membranes with a higher percentage of PCL (See Table 2).

The results of turbidity and conductivity tests are also shown in Table 2. According to the World Health Organization, turbidity is an extremely useful indicator of water quality, because it indicates the presence of suspended chemical and biological particles [39]. In drinking water, a value higher than 5 nephelometric turbidity units (NTUs) compromises water safety and its aesthetic appearance for consumers. Conductivity is also important, as it is routinely used in many industrial and environmental applications as a fast, cheap, and reliable way to measure ionic content in a solution and is directly linked to the amount of total dissolved solids. According to the results, both turbidity and conductivity were reduced considerably (by almost 100% in both cases) by all of the composite PCL/CNF electro-spun membrane systems. This is explained by the fact that the contaminant particles in the tap water are of a larger size than the porous structures of the membranes and, therefore, are easily retained by their pores.

**Figure 7:**
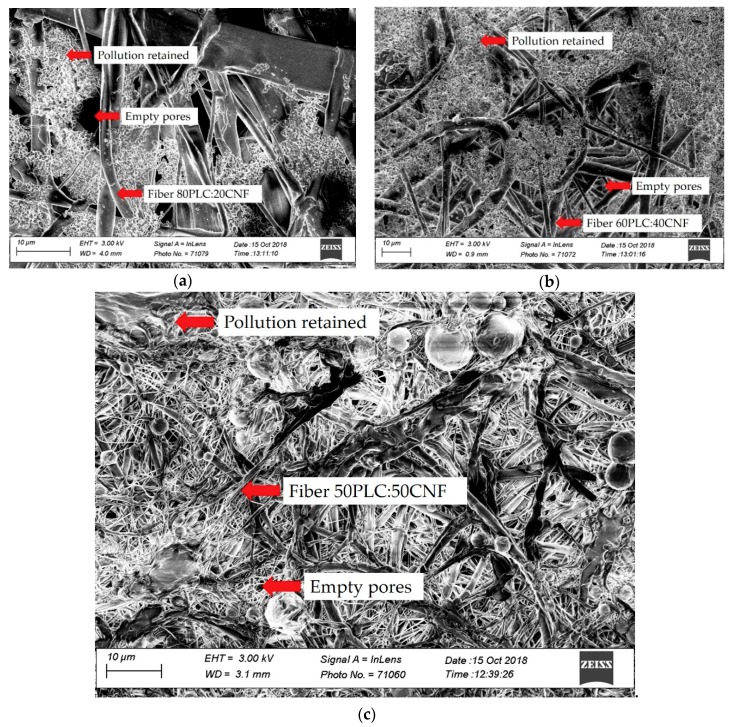
SEM images after filtration of tap water using electro-spun membranes of PCL: CNF (*v*/*v*) ratios: (**a**) 80:20, (**b**) 60:40, and (**c**) 50:50.

SEM was used to analyze the membrane surfaces after filtration. As shown in Figure 7, the contaminating particles present in tap water were adhered to the PCL/CNF electro-spun membranes. In the case of the membrane comprising the PCL80:CNF20 blend, Figure 7a shows areas that are free of contaminating particles; this may be due to the pore size of the membrane being significantly larger than the particle diameter. This could also be related to its lower efficiency in removing heavy metals, as shown in Figure 6. 

In the case of the PCL/CNF (60:40) membrane (Figure 7b), it also shows zones that are free of contaminating particles; they can be seen to have been adsorbed but in a smaller proportion. On the other hand, the contaminating particles fully cover the PCl/CNF (50:50) membrane surface (Figure 7c), showing the best results for removal of heavy metals and greater resistance to tension. Therefore, they are a suitable candidate for use in a filtration system for the purification of contaminated water. 

## 3. Materials and Methods 

Polycaprolactone (molecular weight = 80,000, GPC and melt viscosity ASTM D1238:73); *N*-dimethylformamide (DMF) (anhydrous, 99.8% *w*/*v*); and chloroform (99.8% *w*/*v*) were purchased from Sigma-Aldrich (St. Louis, MO, USA) [40]. Cellulose and nanofiber fibers (CNF) were obtained according to the methodology described in a previous work [7]. In summary, the agave bagasse fibers of the *Agave tequilana* Weber Plant (Arenal Jalisco, Mexico) were delignified, degreased, and cut, and the cellulose was then obtained using the organosolv method. The cellulose pulp was then bleached. Subsequently, agave cellulose fibers were processed at 1.5% consistency in an M-110P microfluidizer and, thus, fibrillation and CNF formation occurred. 

The reagents used to determine the chemical composition were supplied by Sigma-Aldrich (Toluca, Mexico), i.e., aqueous solutions of acetic acid (CH_3_COOH 99% *w*/*v*); sodium chlorite (NaClO_2_, 80% *w*/*v*); potassium hydroxide (KOH, 90% *w*/*v*); sodium hydroxide (NaOH, 97% *w*/*v*); and nitric acid (H_3_NO_3_, 99.5% *w*/*v*). All chemicals were used without further purification.

### 3.1. Preparation of Membranes by Electro-Spinning

For the preparation of PCL/CNF composite membranes, 2 g of CNF was weighed with a consistency of 2.5% *w*/*v* and diluted in DMF. The solution was left in a shaker for 24 h and then placed in ultrasound for 30 min, followed by mixing with PLC (40% *w*/*v*) diluted in chloroform. Three types of membranes were prepared using different PCL:CNF ratios, i.e., 80:20, 50:50, and 60:40 (*v*/*v*). 

The electro-spinning equipment was set up as follows: collector distance, 20 cm; flow rate, 0.5 mL/h; and the voltage 15 to 20 kV (GAMMA, High Voltage Research, FL, USA). The CNF/PCL composite blend was loaded into a 3 mL syringe with a stainless-steel needle and was injected and collected on a plate with a ground connection covered with aluminum foil. After 90 min, the deposited fibers matted into an electro-spun membrane of 10 × 8 cm, which was stored in a dissector for subsequent characterization and use.

### 3.2. Characterization of the Obtained Membranes 

The functional groups in the membranes were analyzed using Fourier transform-infrared (FT-IR) spectroscopy (Nicolet 4700 ATR FT-IR, Thermo Scientific, Grand Island, NY, USA). Sixteen scans were performed in the wavenumber range of 400 to 4400 cm^−1^ and at a resolution of 4 cm^−1^. The membrane crystallinity index was determined based on the empirical method of Segal [7], using X-ray diffraction spectroscopy (XRD; Empyrean, Siemens, Washington, DC, USA). To study the thermal degradation characteristics of the membranes, thermogravimetric analysis (TGA) was carried out (Q100, TA Instruments, Lindon, UT, USA) with heating and cooling rates of 20 °C/min. A 5 mg sample was heated to temperatures in the range of 50 to 400 °C in a nitrogen-rich atmosphere. A flow rate of 20 mL/min was used, and measurements were carried out in duplicate. 

A scanning electron microscope (SEM; Leo 1530-FE, Zeiss, Cambridge, UK) was used to analyze the morphology of the membranes and the contaminant retention capacity. To perform these tests, images of the membranes were taken before and after filtration of contaminated water. The micrographs were obtained at an acceleration voltage of 15 and 20 kV and an increased range of 1000X to 5000X. The ImageJ program was used to measure the diameter of the fiber and porosity area in the SEM image for each membrane [41]. The porosity calculation was according to Equation (1), where *ve* is the volume of empty spaces and *v* is the total volume of the sample:

(1) $$Porosity=\frac{ve}{v}$$

Tensile properties were measured on 0.5-mm-thick, 12-mm-wide, and 50-mm-long strips of the membranes using a universal testing machine (Instron 5967, 50-N load cell) at a crosshead speed of 5 mm/min. The measurements for each sample were carried out in triplicate. Young’s modulus was calculated by dividing the tensile stress by the engineering extensional strain in the elastic (initial, linear) portion of the stress–strain curve. In the case of the electro-spun prepared membranes, their permeability was determined using the Tappi T460 and Gurley method [42], which is based on the time that a volume of air (100 mL) passes through the membrane at a determined and controlled pressure. A Gurley-Hill-type apparatus was used, and the results were expressed in seconds per 100 mL. The calculated permeability was found by using Equation (2):

(2) $$P=\frac{1.27}{t}$$

where *P* is permeability µm/Pa/s and *t* is time in seconds per 100 mL.

### 3.3. Filtration Performance of the PCL:CNF Composite Electro-Spun Membranes

The PCL/CNF membranes were tested for their ability to filter tap water. The tap water was analyzed for certain water quality parameters before and after being filtered with the prepared membranes. The conductivity (Hach model HQ40d, Ocotlán, Jal, México); turbidity (HANNAH Instruments model HI 03703, Ocotlán, Jal, México); and heavy metal concentration atomic absorption spectrometry (ICP Perkin-Elmer—Optima 4300 DV, Madison, WI, USA) were measured. The initial characteristics of the tap water samples are shown in Table 3.

**Figure 8:**
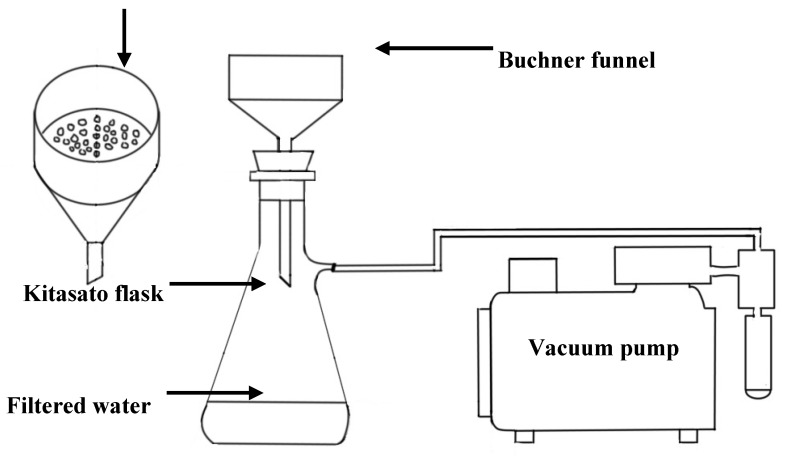
Apparatus used to evaluate water filtration performance of CNF/PCL membrane.

The filtration testing system apparatus consisted of a filtration Buchner funnel that contained the PCL/CNF membrane as a water filter. The funnel was mounted in a Kitasato flask and connected to a vacuum pump (WELCH 1HP, Ocotlán, Jal, México) with a pressure of 59,984.4 Pascal, as shown in Figure 8. The volume of tap water to be filtered was 100 mL, and the time was 10 s. The membranes had a diameter of 4 cm and a thickness of 0.25 mm. After filtering the water, samples of the filtered water were taken and heavy metals, conductivity, and turbidity were measured. The membranes were dried at room temperature, and then SEM photographs were taken and the surface contaminants analyzed.

## 4. Conclusions

Nanocellulose and polycaprolactone composite membranes were developed via electro-spinning. The results of the characterization of the PCL/CNF composite membrane showed a good compatibility between the CNF and PCL, since the mechanical properties of the composite biomaterial were higher, which is important in water filtration systems. Three membrane systems were compared in terms of their ability to improve water quality, in terms of turbidity, conductivity, and the removal of contaminating metals (iron and chromium). The differences found in these properties were interpreted with respect to the nanostructural morphology of the membranes and the interconnection between the composite nanofibers of PCL and CNF. The electro-spun PCL50:CNF50 membrane successfully retained heavy metals in tap water (Fe = 75% and Cr = 99%) and removed 100% turbidity and conductivity. The results showed that CNF is a suitable biowaste material derivative from tequila production, with an added value in the production of composite PCL/CNF electro-spun membranes that can be applied in eco-friendly filtration systems for water purification.

## Figures and Tables

**Table 1:** Morphology of electro-spun nanofibers constituting the membranes. PCL = polycaprolactone and CNF = cellulose nanofiber.

Membrane	Fiber Diameter (nm)	Permeability (µm/Pa/s)	Porosity
PCL80:CNF20	1049 ± 98	0.635 ± 0.01	0.455 ± 0.03
PCL60:CNF40	1747 ± 153	0.317 ± 0.02	0.468 ± 0.02
PCL50:CNF50	216 ± 12	0.317 ± 0.04	0.302 ± 0.05
Commercial cellulose membrane	938 ± 20	1.270 ± 0.04	0.592 ± 0.01

**Table 2:** Reduction of turbidity, conductivity, and heavy metal content (iron and chromium) in tap water before and after filtration.

Membrane	Turbidity (NTU)		Conductivity (S/m)		Iron Reduction (%)	Chromium Reduction (%)
	Before	After	Before	After		
PCL80:CNF20	70 ± 3.5	0.91	669 ± 3.5	0	38 ± 1.9	42 ± 2.1
PCL60:CNF40	83 ± 4.1	0.00	669 ± 3.5	0	62 ± 3.1	90 ± 4.5
PCL50:CNF50	83 ± 4.1	0.00	669 ± 3.5	0	75 ± 3.5	99 ± 3.5

**Table 3:** Characteristics of the tap water used for testing filtration performance of the electro-spun membrane.

pH	Temperature (°C)	Conductivity (μS/cm)	Turbidity (NTU)	Heavy Metal Concentration (ppb)		
				Chlorine	Iron	Chromium
8.69 ± 0.17	27.5 ± 0.5	669 ± 0.18	81 ± 1.62	57.041 ± 0.11	6.676 ± 0.13	0.432 ± 0.08
